# Plasma-Based Genotyping in Advanced Solid Tumors: A Comprehensive Review

**DOI:** 10.3390/cancers13215299

**Published:** 2021-10-22

**Authors:** Maisam Makarem, Miguel García-Pardo, Natasha B. Leighl

**Affiliations:** Princess Margaret Cancer Center, Department of Medical Oncology, Toronto, ON M5G 2C1, Canada; maisam.makarem2@uhn.ca (M.M.); miguel.garcia@uhn.ca (M.G.-P.)

**Keywords:** plasma genotyping, ctDNA, guideline recommendations

## Abstract

**Simple Summary:**

Targeted therapy is at the forefront of cancer diagnosis and treatment today for multiple advanced tumors. Although molecular testing of tumour tissue biopsies remains the gold standard for molecular diagnosis, it has certain limitations. There have been major advances in the use of plasma, also referred to as a “liquid biopsy,” to identify changes in the genome associated with approved targeted therapies. Here, we review key studies that have led to these approvals and a paradigm shift toward greater use of liquid biopsy in precision oncology.

**Abstract:**

Molecular genotyping for advanced solid malignancies has transformed the clinical management of patients with metastatic disease. Treatment decisions in a growing number of tumors require knowledge of molecularly driven alterations in order to select optimal targeted therapy. Although genomic testing of tumor tissue is the gold standard for identifying targetable genomic alterations, biopsy samples are often limited or difficult to access. This has paved the way for the development of plasma-based approaches for genomic profiling. Recent advances in the detection of plasma-circulating tumor DNA (ctDNA) have enabled the integration of plasma-based molecular profiling into clinical practice as an alternative or complementary tool for genomic testing in the setting of advanced cancer, to facilitate the identification of driver mutations to guide initial treatment and diagnose resistance. Several guidelines now recommend the use of plasma where tumor tissue is limited to identify a targetable genomic alteration. Current plasma-based assays can evaluate multiple genes in comprehensive panels, and their application in advanced disease will be increasingly incorporated into standard practice. This review focuses on current and future applications of plasma ctDNA-based assays in advanced solid malignancies, while highlighting some limitations in implementing this technology into clinical practice.

## 1. Introduction

The characterization of a variety of genomic aberrations driving solid tumor growth and the subsequent development and utilization of targeted therapies has been associated with improved clinical outcomes for patients with advanced and early-stage disease, leading to significant changes in the standard of care [1]. Despite these advances, resistance inevitably develops over time, altering successive treatment options for patients. Therefore, identifying new genomic targets through various stages of a patient’s treatment trajectory becomes an essential component of practice [2]. 

Pathologic analysis of tumor tissue, derived from surgical resections or biopsy specimens, remains the standard method for confirming a histologic cancer diagnosis. However, limitations exist for extensive genomic analysis, relating to tissue quantity and requirements for re-biopsy. Using only tumor tissue for genotyping can lead to more invasive procedures such as rebiopsy with the associated cost and longer turnaround times delaying treatment initiation [3,4]. The invasive nature of repeat tissue biopsy also precludes serial sampling required to understand tumor clonal dynamics. Furthermore, a tumor tissue biopsy may not represent the genomic heterogeneity of the primary tumor or metastatic disease sites.

Liquid biopsies have emerged as a practical tool to analyze molecular alterations in the tumor through the isolation of cell-free DNA, of which circulating tumor DNA (ctDNA) makes up a small proportion. This has presented a minimally invasive method to isolate various analytes, including DNA, RNA, circulating tumor cells (CTCs), tumor-educated platelets and exosomes, among others [5]. Several bodily fluids, including blood (plasma, serum), urine, saliva, pleural, and cerebrospinal fluid, have been evaluated as noninvasive surrogates of tumor monitoring, with plasma being preferred [6,7]. Liquid biopsies also allow for the evaluation of the patient’s cancer at multiple time points during treatment, providing insight into real-time dynamics of the tumor in response to therapy. ctDNA can also originate from multiple tumor sites, delivering more comprehensive information about tumor heterogeneity compared to single-site tissue biopsies [8]. Some limitations exist, including lower sensitivity with false-negative results seen in up to 30% of cases, the short half-life of ctDNA in the bloodstream (30 min to 2 h), and a lack of standardized pre-analytical, analytical, and reporting methods [9,10]. These may vary by cancer subtype and require demonstration of clinical validity for use. In addition, recent advances in rare cell isolation technologies and affordable technologies such as next-generation sequencing (NGS) and droplet digital polymerase chain reaction (ddPCR) have produced promising clinical results, allowing the application of plasma-based genotyping in the routine management of patients with solid malignancies [11,12,13].

This article provides an overview of current and future applications of plasma ctDNA-based assays in select advanced solid malignancies, including recent guidelines and recommendations. The review focuses on breast, prostate, ovarian, lung, and colorectal malignancies, but the application of plasma-based testing extends to several other solid tumors and has been highlighted in recent comprehensive reviews [14,15,16,17,18]. In addition, the results of recent trials demonstrating the clinical utility of plasma ctDNA assays are interpreted in the context of current clinical practice and the evolving treatment landscape for solid tumors.

## 2. Clinical Utility of Plasma-Based Genotyping

Several studies have explored the effectiveness of detecting targetable molecular alterations in plasma compared with tumor tissue-based genotyping. Most studies have shown a high concordance between the two and have demonstrated the added value of plasma in detecting actionable targets. In the NILE study, the ability of a validated, comprehensive plasma ctDNA test to identify guideline-recommended biomarkers was demonstrated to be noninferior to standard-of-care tissue genotyping in patients with advanced nonsmall cell lung cancer (NSCLC). Plasma testing identified actionable genomic targets for 27.3% of patients compared to 21.3% by tissue testing (*p* < 0.0001), with a faster turnaround time (median 9 vs. 15 days). The addition of plasma genotyping increased the rate of identification of a guideline-recommended genomic biomarker by 48% (from 60 to 89 patients) [12]. In another study of 264 patients with advanced NSCLC, plasma ctDNA NGS testing identified 26% more actionable genetic alterations (48 vs. 38 patients) compared with tumor tissue testing [19], with similar results seen in another prospective cohort study [11].

In contrast to the outcomes described above, a tissue-based multigene NGS panel demonstrated higher sensitivity and accuracy in a study of 100 patients with lung adenocarcinoma compared to a plasma ctDNA-based assay [20]. Within a subgroup of patients with Stage IV disease who had not received treatment, tissue-NGS sensitivity was higher than plasma (93% vs. 63%). Alterations identified by tissue NGS testing but not plasma testing included hotspot mutations in *EGFR*, *BRAF*, and *KRAS*. However, access to tissue can pose major challenges in some cancers. For example, in a study of patients with acquired resistance to EGFR kinase inhibitors, repeat biopsy for genomic analysis was only successful for 81% of patients [21]. Although the sensitivity of plasma ctDNA analysis was 75%, it increased the number of patients eligible for further targeted therapy from 49%, using tissue testing only, to 73% using both plasma and tissue testing.

In the largest prospective cohort of patients with metastatic castrate-resistant prostate cancer (mCRCPC) from the TRITON clinical trials, over 3000 patients underwent plasma testing, which aimed to characterize the genomic landscape of advanced prostate cancer. Of these, 94% had detectable ctDNA. Plasma testing identified *BRCA1/2* alterations in 8.9% of patients, and identified a DNA damage repair gene alteration in a third of samples. Among patients with a *BRCA1/2* alteration, 2.4% of patients had alterations detected only in plasma, some with high variant allele fractions, suggestive of either clonal heterogeneity not captured in tumor tissue, or alterations acquired during treatment after tissue collection. ctDNA was also highly sensitive in detecting androgen receptor-associated resistance mutations, as well as secondary mutations and reversion mutations in the BRCA gene [22]. These findings have the potential to inform subsequent treatment strategies with a greater understanding of emergent resistance mechanisms.

Analysis of circulating tumor cells (CTCs) is also an important method of using plasma testing to direct therapy. In a recent large prospective clinical trial, STIC CTC, patients with hormone receptor-positive, *HER2*-negative metastatic breast cancer were randomized to receive first-line treatment based on investigator choice or a CTC-informed first-line treatment option, to help guide the choice between chemotherapy or endocrine therapy in this setting. The study met its primary endpoint showing noninferiority in progression-free survival in the CTC-informed compared with the clinically selected arm (HR 0.94). In 38.7% of patients, discordant results were present between clinical and CTC assessment (clinical low, CTC high subgroups or vice versa). Patients in the subgroup with clinical low risk but CTC high had a longer PFS with CTC-informed chemotherapy compared with the clinician-selected endocrine therapy arm, with no difference in overall survival (OS) [23].

The results from these studies clearly demonstrate that plasma NGS testing can play an important role in identifying genomic alterations and guiding therapy. However, the variable sensitivity of the assay due to tumor-, sampling-, and testing technique-related factors might influence results, and negative ctDNA NGS results need to be interpreted with caution, especially in the setting of low allele frequencies, and, where possible, confirmed using tissue-based testing methods. The analytical validity of several plasma-based assays has been described in recent articles [24,25].

## 3. Guideline Recommendations and Future Directions

### 3.1. Breast Cancer

Clinical guidelines reflect the clinical utility of plasma-based genotyping for *PIK3CA* in patients with advanced hormone receptor-positive, treatment-resistant breast cancer (Table 1). Although there is a correlation between plasma cell-free DNA levels and advanced disease, there are no current applications for plasma testing in the initial diagnostic setting. Some studies have explored the utility of ctDNA in predicting nodal metastasis, risk of relapse, and survival in patients with breast cancer. However, the development of breast cancer-specific gene panels or bespoke assays may improve the detection of relapsed disease in the future [26,27,28].

The current first-line treatment for patients with metastatic hormone receptor-positive breast cancer includes endocrine therapy in combination with a CDK4/6 inhibitor, such as ribociclib, palbociclib, or abemaciclib. Several trials have shown significant overall survival benefits [37]. However, as with many targeted therapies in solid tumors, resistance develops, and in up to 20% of patients, activation of the PI3K/AKT/mTOR signaling pathway is a leading driver of acquired resistance to therapy.

Concordance rates for detecting genomic alterations in liquid biopsies in patients with metastatic breast cancer in paired tissue and plasma samples have been recently explored using the Guardant360^TM^ assay (Guardant Health, Inc., Redwood City, CA, USA). For *PIK3CA* mutations, there was an 81% observed agreement between tissue and plasma. In 7.6% of cases, PIK3CA gene alterations were detected in tissue but not in plasma, and 11.4% in plasma only [38].

The approval of the therascreen^®^ PIK3CA PCR assay as a companion diagnostic to detect PIK3CA gene mutations in tissue or plasma has brought the use of liquid biopsy as a diagnostic tool closer to patients with breast cancer. This particular assay has been approved based on evidence of clinical utility from SOLAR-1, which demonstrated that patients with metastatic hormone-positive breast cancer progressing on endocrine therapy and plasma ctDNA evidence of a PIK3CA gene mutation derived an overall survival benefit from subsequent treatment with alpelisib plus fulvestrant with a median survival of 34.4 months vs. 25.2 months [39]. In addition, plasma samples from PALOMA-3 showed that early changes in *PIK3CA* ctDNA levels may predict PFS in metastatic breast cancer patients receiving CDK4/6 inhibitors [40].

In the future, the use of plasma testing to predict treatment response to endocrine therapy may also be of benefit to patients. *ESR1* mutations in plasma ctDNA appear in patients who have received endocrine therapy, are highest in patients with metastatic disease [41], and may help guide future therapy. *ESR1* mutations can be detected in plasma at a median of 6.7 months before evidence of clinical progression [42]. In the SoFEA trial, patients with *ESR1* mutations detected in plasma at baseline had improved PFS if they received fulvestrant compared with exemestane [43], which may help inform optimal endocrine therapy. However, in the recent plasma-MATCH Phase II study in a heavily pretreated patient population that assessed the feasibility and utility of ctDNA in directing targeted therapy in breast cancer, fulvestrant demonstrated similar activity in patients with and without ESR1 gene mutations in plasma, suggesting more complex clonal dynamics at play [44].

Several studies are exploring the utility of ctDNA in predicting pathologic complete response after neoadjuvant treatment [45], which is changing the therapeutic landscape of *HER2*-positive and triple-negative breast cancer patients. Other studies are exploring the role of circulating tumor cells (CTCs) as a prognostic tool to improve prediction of clinical outcomes [46].

### 3.2. Prostate Cancer

Metastatic castrate-resistant prostate cancer (mCRPC) remains challenging to treat, with a median OS ranging from 13 to 30 months [47]. Although multiple androgen-signaling inhibitors have shown survival benefits, recent advances utilizing plasma-based testing have come from targeting DNA-repair pathway-related alterations with PARP inhibition.

Recent trials for patients carrying DNA repair pathway-related alterations have demonstrated a role for PARP inhibitors with significant OS benefits. The PROfound trial was a phase 3 trial assessing the efficacy of olaparib versus enzalutamide or abiraterone in men with alterations in *BRCA1/2* or *ATM*, or other homologous recombination repair (HRR) gene mutations, and they showed significant improvements in survival with targeted therapy (19 vs. 14 months) [48]. The cohort of patients with *BRCA1/2* or *ATM* mutations was also tested using the plasma FoundationOne Liquid^TM^ assay, and PFS outcomes were similar to outcomes based on tissue testing [49].

TRITON2 analyzed the role of rucaparib in patients with alterations in HRR genes. Patients with BRCA gene alterations benefited from rucaparib with similar response rates whether the alteration was identified in plasma (46.3%) or in tissue (43.5%) [50]. These studies led to the approval of two PARP inhibitors, olaparib and rucaparib, for patients with mCRCP with *BRCA* alterations using either a tissue or liquid companion diagnostic test. In the largest cohort of patients with mCRPC (N = 3334 plasma samples), including patients from TRITON2 and TRITON3, 94% of patients had detectable plasma ctDNA, and a high level of concordance was seen (>90%) between plasma and tissue identification of *BRCA1/2* mutations, with 93% of mutations identified in tumor tissue detected also in plasma (67/72 patients with *BRCA1/2* mutations) [22]. Relevant genomic alterations also included changes in androgen receptor signaling, including resistance, DNA damage repair, and microsatellite stability, which may predict the response to immune checkpoint inhibitors.

Recent studies have also explored the role of circulating tumor cells (CTCs). CELLSEARCH^TM^ was the first and only FDA-approved assay to date for CTC quantitation by enriching for EpCAM-positive cells in the monitoring of patients with advanced breast, colorectal, and metastatic prostate cancer (Table 1). In the PROPHECY trial, a prospective blinded study of men with high-risk castrate-resistant disease starting abiraterone or enzalutamide, the baseline expression of the *AR-V7* splice variant in CTCs (mRNA or protein) was independently associated with clinical outcomes of progression-free and overall survival, indicating prognostic value [51]. Characterization of the epigenetic changes in CTCs may also be moving into the forefront of discovery of new clinical applications [52].

### 3.3. Non-Small Cell Lung Cancer

In the current era of targeted therapy, treatment decisions in advanced NSCLC require knowledge of molecular alterations in order to select optimal cancer therapy. The detection and characterization of targetable genomic alterations that drive tumor growth has prompted an increasing number of guideline-based recommendations for many actionable targets for drug therapy that have demonstrated improved patient outcomes, including *EGFR*, *ALK*, *ROS1*, *BRAF*, *MET*, *RET*, or *HER2* [33,53,54]. The European Society of Medical Oncology (ESMO) recommends routine use of broad-panel NGS on tumor samples in patients with advanced non-squamous NSCLC and other solid tumors [55]. However, tissue biopsy and NGS testing are not always feasible, given the long turnaround times and the invasive modalities required for access (EBUS or CT-guided biopsy) in patients who are clinically unwell, and tissue may be insufficient to test all relevant targets [12,56]. Thus, plasma ctDNA testing has significant potential to improve molecular genotyping and access to precision medicine in patients with advanced NSCLC.

Several studies in lung cancer, as well as other solid tumors, have demonstrated excellent concordance between plasma ctDNA analysis for molecular genotyping with tissue profiling. Recent data from patients with *ALK* fusion-positive disease detected in plasma and treated with alectinib had a high response rate and PFS, similar to outcomes with registration trials that used tissue testing [57]. Similarly, there were no differences in PFS or response rate with EGFR kinase inhibitors in patients with *EGFR* mutations detected in plasma compared to tissue testing, and also no differences by low or high variant allele frequencies [58].

A growing number of studies of novel targeted agents such as capmatinib and tepotinib for *MET* alterations have also used liquid biopsy as a diagnostic method for target identification in patients, leading to broader regulatory acceptance of plasma ctDNA testing as a companion diagnostic in patients with advanced NSCLC [59,60]. Moreover, the Canadian VALUE study (NCT03576937) showed that routine plasma ctDNA testing in addition to standard-of-care tumor tissue genotyping resulted in more patients accessing targeted therapy compared to standard tissue testing alone, with 37% of treatment decisions being informed by plasma testing [61]. Plasma testing may also lead to cost savings, with more patients with oncogene-addicted cancers accessing targeted therapy and fewer patients accessing more expensive and less effective checkpoint inhibitor therapy [62].

All these studies have validated the clinical utility of plasma-based NGS as an additional method to guide precision medicine in advanced NSCLC patients at diagnosis. Plasma-based NGS is rapidly being integrated into clinical practice for molecular genotyping in patients with advanced NSCLC. Plasma testing is also preferred for patients who are medically unfit for repeat tumor biopsy. Moreover, the International Association of the Study of Lung Cancer (IASCLC) consensus statement in 2021 concluded that plasma ctDNA approaches have significant potential to improve patient care, and immediate implementation in the clinic is justified in a number of therapeutic settings in NSCLC, including in the diagnosis of EGFR kinase inhibitor resistance (*EGFR* T790M) [6]. For patients with acquired resistance to targeted therapy, a plasma-first approach is recommended with reflex to tissue biopsy if results are negative or histologic transformation is suspected. In the setting of patients with treatment-naïve advanced nonsquamous NSCLC, liquid biopsy is encouraged as a serial, complementary, or plasma-first approach, in addition to standard tumor tissue molecular profiling, again with reflex to tissue in the case of a negative initial plasma test [6].

Additionally, given the major impact of wait times for molecular testing results on patient outcomes, liquid biopsy has emerged as a potential method to accelerate the molecular diagnosis and time to treatment of patients with advanced lung cancer. It is currently being investigated through prospective trials in patients with radiographic evidence of advanced lung cancer, prior to diagnostic tissue biopsy and profiling. These studies aim to shorten the time to treatment for patients with and without targetable alterations (ACCELERATE, NCT04863924; [63]).

Finally, targeted therapy is moving to the curative setting, with trials such as ADAURA showing a disease-free survival benefit among patients with resected stage IB-IIIA *EGFR*-mutation-positive NSCLC treated with adjuvant osimertinib [64]. In patients with stage III *EGFR*-mutated NSCLC, the role of consolidation durvalumab after chemoradiation is unclear, with some preliminary data suggesting the possible lack of benefit and higher frequency of adverse events [65]. Therefore, potential applications of ctDNA in NSCLC are not limited to the advanced disease setting, and may soon become integrated into the management of early-stage disease.

## 4. Colorectal Cancer

In metastatic colorectal cancer (mCRC), *KRAS*, *NRAS*, and *BRAF* assessment is mandatory for treatment selection and prognostication, as *RAS* mutations confer resistance to anti-EGFR antibodies and *BRAF* V600 mutations indicate poor prognosis. NGS testing for extended RAS and BRAF gene mutations is currently standard practice, and ctDNA testing using single gene assays (PCR-based) has been shown to be an effective alternative to tissue-based genotyping and is approved by the European Medicines Agency (Idylla^TM^ ctKRAS, Idylla^TM^ ctNRAS-BRAF, OncoBEAM^TM^ RAS) but remains to be incorporated into clinical guidelines [66,67,68,69,70,71,72,73,74,75,76,77,78]. Although tumor tissue in patients with metastatic CRC is often easier to obtain than in lung cancer patients, the benefits of fast turnaround time with plasma ctDNA ddPCR testing may confer a benefit to patients needing to start treatment sooner.

The concordance between plasma and tissue for RAS gene mutation testing is approaching 90% and is similar for BRAF gene testing. Using OncoBEAM^TM^ RAS CRC, 280 Asian patients with metastatic CRC had a high concordance of plasma and tissue-based analysis (86.4%), and concordance rates between BEAM-ing and NGS for plasma testing were 96%. Interestingly, the concordance in patients with lung metastases alone was lower (64.5%), perhaps indicating the clonal heterogeneity of these metastases [69]. Another prospective real-world analysis found a high percent agreement in plasma and tissue RAS gene mutation testing (mCRC) with a concordance of 89% using BEAMing [70].

With respect to clinical outcomes, the identification of a mutation in plasma appears to correlate with similar outcomes as compared to identifying the alteration in tissue. In a retrospective analysis of a subset of patients with mCRC from the CAPRI-GOIM trial, plasma *RAS* testing by ddPCR showed a 78.3% concordance with tissue NGS, and similar PFS and survival outcomes with cetuximab plus FOLFIRI whether RAS gene mutations were identified in tissue or plasma [71].

Currently, several trials are exploring the clinical utility of ctDNA analysis in mCRC in different settings. The recent CHRONOS trial (NCT03227926) explored the role of ctDNA analysis to select patients for rechallenge with panitumumab in the third-line setting in patients with *RAS* wildtype mCRC. Of 52 patients tested, 69% had no ctDNA evidence of *RAS/RAF/EGFR* mutations. Rechallenge panitumumab was associated with a response rate of 30%, demonstrating the potential of ctDNA to identify patients for successful retreatment [72].

Moving forward, in early-stage colorectal cancer (CRC), there is compelling evidence demonstrating the role of ctDNA detection after curative-intent therapy as an important prognostic factor [73]. This approach, known as minimal residual disease (MRD) detection, represents an opportunity to better select patients that may benefit from more intense adjuvant therapy, or those that do not need further chemotherapy, with the aim of improving survival rates and minimizing toxicity and unneeded therapy [74].

Investigators have used a range of fixed and personalized tumor-informed panels (e.g., Signatera^TM^ bespoke multiplex PCR NGS assay) to track tumor-specific single nucleotide variants (SNVs). Tie et al. used the presence of post-operative ctDNA using an individualized panel to select patients for adjuvant therapy after resection of stage III colon cancer [75]. Patients underwent plasma ctDNA testing at 4–10 weeks after surgery. If ctDNA was detected, patients received adjuvant chemotherapy. Patients had repeat ctDNA testing 6 weeks after completing chemotherapy, and if they experienced ctDNA clearance, their estimated 3 year relapse-free interval was 77% versus 30%. A larger randomized trial testing the impact of adjuvant chemotherapy on RFS in patients with plasma ctDNA MRD after colon cancer resection has completed accrual and the results are pending (NCT04058103), as are the results of other studies. We anticipate that ctDNA detection will play an important role for MRD detection and adjuvant therapy decisions in early-stage CRC and other solid tumors in the near future.

### Ovarian Cancer

The role of liquid biopsy in ovarian cancer has focused on predicting the response to treatment and detecting resistance mutations. Clinical management has relied on serum CA125 as a key biomarker in the advanced disease setting, with certain pitfalls owing to its poor sensitivity and specificity [76,77]. To date, no clear driver oncogene has been identifiable given the widely heterogenous biology of these tumors, but *BRCA1/2* mutations and HRR-related gene alterations have emerged as clinically-relevant genomic aberrations in patients with high-grade serous ovarian cancer [78]. Approximately 25% of these patients harbor a pathogenic *BRCA1/2* mutation, which is associated with a favorable response to PARP inhibitors.

To date, several trials have demonstrated the benefit of PARP inhibitors as a first-line maintenance phase of therapy for patients with platinum-sensitive high-grade *BRCA*-mutated ovarian cancer (SOLO1, [79]), as second-line maintenance therapy in relapsed disease irrespective of BRCA gene status (SOLO2, ARIEL3, [80,81]), and in the recurrent setting as monotherapy in heavily pretreated, PARP-inhibitor naive, *BRCA* mutant ovarian cancer patients (SOLO3, [82]).

Currently, the US FDA has approved NGS testing in ovarian cancer using several companion diagnostics, including FoundationOne^TM^ CDx (includes HRR genes), myChoice^®^ CDx, and in the liquid biopsy space, approval for FoundationOne^®^ Liquid CDx to the direct use of rucaparib after 2 or more lines of chemotherapy, based on results from the ARIEL-2 trial. The trial evaluated the concordance of liquid and tissue-based testing, as well as the clinical efficacy of rucaparib in patients carrying *BRCA1/2* alterations in plasma ctDNA [83]. Patients with somatic or germline *BRCA1/2* gene mutations who were enrolled in the ARIEL2 clinical trial had plasma ctDNA collected before and after rucaparib treatment. The concordance between plasma ctDNA and tissue had a high positive percent agreement (93.8%) and negative percent agreement (97.4%) with similar clinical efficacy [84].

Plasma-based assays have also been used to identify reversion mutations in patients whose cancer may not respond well to PARP inhibition. The development of *BRCA* gene reversion mutations, reactivating wild-type protein activity, has been a key mechanism of resistance to platinum-based chemotherapy and PARP inhibitors. An assessment of 121 patients with high-grade serous ovarian cancer by NGS in ctDNA showed similar frequencies of germline, somatic, and reversion mutations [85]. Importantly, the absence of reversion mutations in ctDNA after platinum-based therapy is associated with longer PFS with subsequent rucaparib treatment (HR 0.12) [86]. In addition, the detection of reversion mutations in plasma preceded radiologic progression by a median of 3.4 months. Detection of these reversion mutations presents a valuable clinical tool to predict the response to PARP inhibition. The limitation is that this phenomenon is uncommon, with 7% of patients identified to have a reversion mutation in ctDNA pre-rucaparib treatment and another 7% identified post-progression [86]. Weigelt et al. identified 4 out of 19 patients (21%) as having polyclonal *BRCA1/2* reversion mutations using cell-free DNA from patients with platinum refractory or resistant ovarian cancer [87].

The use of multimodal cell-free DNA assays may also help to predict treatment response more reliably than CA-125. For example, the detection of methylated ctDNA at the promoter of *HOXA9* (previously shown to be a poor prognostic factor) has been associated with poorer clinical outcomes in patients with platinum resistant ovarian cancer, with a median OS of 9.5 months vs. 19.4 months in patients without detectable methylation in *HOXA9* [88].

Several studies have also explored the use of changes in allelic frequencies of TP53 gene variants as a marker of treatment response. TP53 gene mutations are the most common pathogenic mutation in solid tumors, as well as high-grade serous ovarian cancer (HGSOC). In a retrospective analysis, Parkinson et al. showed that in patients with relapsed HGSOC, a larger decrease (>60%) in *TP53* variant allele frequency during chemotherapy was associated with a longer time to progression [89]. They also showed that allelic fractions of *TP53* variants in plasma were associated with tumor volume, and the predicted response to treatment was earlier than CA125 levels [89]. In addition, Piskorz et al. looked at the feasibility of monitoring treatment response in the ARIEL2 study using the *TP53* variant allele fraction. Similar to the study by Parkinson et al., a greater decrease in *TP53* mutant allele fraction (greater than 50%) was associated with the response to treatment [90]. Thus, monitoring levels of *TP53* mutations by plasma ctDNA analysis could represent a novel approach to monitoring treatment response over time.

## 5. Limitations of Plasma ctDNA Testing in Clinical Practice

Although liquid biopsy has several advantages in genotyping of metastatic solid tumors, there are limitations that can be encountered. These can occur in the pre-analytical phase, i.e., from the time of sample collection, and in the analytical and post-analytical phases, i.e., during result interpretation owing to technical considerations, as well as tumor biology, among other factors. The goal is to have an analyte that reflects tumor heterogeneity while minimizing false negative and false positive results. The quantity of ctDNA in plasma can directly influence the ability to detect specific allelic mutations. Very low levels of specific variants may not be detectable by existing assays. Therefore, a lower burden of disease or metastatic spread, or in cases where disease is limited to the CNS or other sanctuary sites, may require more sensitive assays to detect genomic variants that may be present at lower allelic frequencies, making it more likely that patients will require tissue biopsies for accurate genotyping results [8].

This has greater implications in the setting of MRD, post-neoadjuvant treatment, or in the adjuvant treatment setting [91]. Recent technologies such as CAPP-Seq [92] and others [93] will offer significant advantages in settings of low variant allele frequency. In addition, the use of tumor-informed assays may also increase the ability to detect MRD and predict recurrence in a more sensitive manner [94].

Many ctDNA genotyping platforms have demonstrated high specificity and positive predictive value (PPV) for detecting mutations of interest, although lower PPV may be a challenge in situations where variant allele frequencies are low [25]. This increases the risk of false positives being detected, with non-tumor-related mutations being contributed from clonal hematopoiesis (CHIP), impeding interpretation. CHIP-related gene alterations have included *TET2*, *TP53*, *DNMT3A*, and *JAK2*, as well as actionable targets such as *KRAS*, highlighting the importance of identifying tumor-specific alterations [95]. In a recent case series of patients with prostate cancer, a high proportion of ATM gene variants were present in plasma attributable to CHIP, impacting the ability to identify patients eligible for PARP inhibitors and the association with clinical efficacy [96]. Some of this can be circumvented by the incorporation of genotyping of matched patient leukocytes as a control.

Although early ctDNA assays may have been more limited in their detection of gene fusions and copy number alterations, several panels now have an increased sensitivity of detection of genomic alterations such as fusions or copy number alterations, and bioinformatic advances have also improved fusion calling algorithms. In the NILE study, assay improvements allowed *ALK* fusions to be detected with increased sensitivity and 100% PPV [12]. A recent analysis exploring the potential to detect NTRK-fusions in plasma, which have several tumor-agnostic FDA drug approvals, has also shown to be feasible with high PPV [97].

The clinical utility of ctDNA testing is growing for patients with oncogene-addicted tumors where targeted therapy is the standard of care. However, for patients with non-oncogene-driven tumors, ctDNA testing is still not ready for clinical use, although there are some interesting biomarkers under study. For example, in lung cancer, the quantification of tumor programmed cell death ligand-1 (PD-L1) expression to direct checkpoint inhibitor therapy requires tissue immunohistochemistry testing. Other potential biomarkers such as tumor mutation burden (TMB) can be quantified using plasma as a surrogate for tissue TMB, and some studies have shown improved clinical outcomes in patients with high plasma TMB levels and a response to checkpoint inhibitors [98]. However, further clinical validation is needed prior to implementation in practice. Lastly, plasma-based testing offers limited insight into histologic cellular transformation events, which can occur in 5–15% of patients receiving targeted therapy for lung cancer [99,100].

## 6. Conclusions

Plasma genotyping for multiple solid tumors is currently being implemented into clinical practice (Table 1), including the US FDA approval of several companion diagnostic tests. Currently, these offer a select panel of targetable genomic alterations for detection, and in the presence of actionable mutations found in plasma, the treating physician can offer targeted therapy. This is the case for *PIK3CA* gene mutations in breast cancer, several genomic targets in lung cancer, including in *EGFR*, *ALK*, *ROS-1*, *BRAF*, *MET*, *RET*, *KRAS*, and *NTRK*, and *BRCA1/2* alterations in prostate cancer. The role of plasma ctDNA testing will continue to expand, improving patient access to precision medicine and minimizing invasive tissue biopsies.

The future of cancer therapy depends on the identification of oncogenic drivers, as well as epigenomic, transcriptomic, and other nononcogene biomarkers. Incorporating these into current assays and patient diagnostic workflows will be critical. Although we are not ready for the use of ctDNA in disease monitoring and MRD detection, tumor-informed assays are showing promise and may lead to better patient outcomes, as well as de-escalation of therapy where appropriate, and cost savings. Our understanding of the limitations of this technology and standardizing pre-analytical, analytical, and interpretation processes are critical to ensuring high-quality patient care. We look forward to the results of ongoing and future trials that demonstrate the clinical utility and cost-effectiveness of this technology not only at initial diagnosis of advanced disease but throughout the cancer journey.

## Figures and Tables

**Table 1:** Summary of selected guideline recommendations, US FDA approvals.

	FDA-Approved Diagnostic Plasma ctDNA Tests	ESMO	ASCO	NCCN
Breast Cancer	therascreen PIK3CA RGQ PCR Kit FoundationOne^®^ Liquid CDx (*PIK3CA* mutations)	ctDNA assessment not recommended for disease monitoring, detection of progression in advanced breast cancer. *PIK3CA*: ctDNA testing is an option for detection of *PIK3CA* mutations for selecting patients for alpelisib therapy. Reflex to tissue testing if ctDNA uninformative ESR1: *ESR1* mutation status assessment not ready for routine clinical use [29]	*PIK3CA*: Cell-free DNA detection of *PIK3CA* mutations is recommended. Reflex to tissue if no mutation detected. *ESR1:* Routine testing unlikely to affect treatment decisions [30]	*PIK3CA*: Mutation testing can be performed on tumor tissue or ctDNA in peripheral blood. If ctDNA-negative, reflex to tissue is recommended. CTC: No recommendation given lack of predictive value (SWOG0400 [31])
Ovarian	FoundationOne^®^ Liquid CDx (*BRCA1/2* for rucaparib)	Quantification of cell-free DNA is not established, to assess for disease response and relapse [32]	No formal recommendations for plasma-based testing	No formal recommendations for plasma-based testing
Prostate	FoundationOne^®^ Liquid CDx (*BRCA1/2*, *ATM*) for rucaparib, olaparib	No formal recommendations for plasma-based testing	No formal recommendations for plasma-based testing	HRR * gene alterations: Metastatic tissue biopsy preferable. If not possible, plasma ctDNA testing is an option at the time of biochemical or radiographic progression.
Lung (non-small cell lung cancer) **	FoundationOne^®^ Liquid CDx (for ALK gene rearrangements, EGFR gene mutations, MET gene exon 14 skipping mutations) cobas EGFR Mutation Test v2 (plasma, for *EGFR* exon 19 or 21 mutations eligible for approved TKI) Guardant360^®^ CDx (*EGFR* exon 19 deletions, L858R, and T790M, exon 20 insertions for amivantamab, *KRAS*G12C for sotorasib)	*EGFR* plasma testing can be considered before tissue testing to detect T790M. If negative, tissue biopsy is recommended [33].	When tissue is limited, cell-free DNA to identify EGFR T790M gene mutations is recommended. If plasma-negative, tissue testing advised [34].	Consider if the patient is medically unfit for invasive tissue sampling, or if insufficient material after cancer diagnosis for molecular analysis (EGFR, KRAS, ALK, ROS1, BRAF, NTRK, MET, and RET gene alterations). Plasma testing should be considered at progression on EGFR TKIs to assess for T790M
Colorectal	No current FDA approved companion diagnostic tests	ctDNA and CTC are not recommended in routine practice [35]	Clinical application of liquid biopsy requires further validation ***	No formal recommendations for plasma-based testing

CTC: Circulating tumor cells. * Homologous recombination gene mutations to be considered for use of olaparib include *BRCA1*, *BRCA2*, *ATM*, *BARD1*, *BRIP1*, *CDK12*, *CHEK1*, *CHEK2*, *FANCL*, *PALB2*, *RAD51B*, *RAD51C*, *RAD51D*, and *RAD54L*. ** IASLC consensus statement recommendations are concordant with stated recommendations [6], including ctDNA testing for initial genotyping of advanced disease using a plasma-first, sequential or complementary approach to tissue genotyping, and a plasma-first approach for resistance to targeted therapies. *** Combined ASCP, CAP, AMP, and ASCO guideline recommendations for molecular biomarker evaluation [36].
